# Evolutionary and plastic responses of freshwater invertebrates to climate change: realized patterns and future potential

**DOI:** 10.1111/eva.12108

**Published:** 2013-10-10

**Authors:** Robby Stoks, Aurora N Geerts, Luc De Meester

**Affiliations:** Laboratory of Aquatic Ecology, Evolution and Conservation, University of LeuvenLeuven, Belgium

**Keywords:** aquatic insects, experimental evolution, hydroperiod, latitudinal gradients, rapid evolution, salinity, space-for-time substitution, zooplankton

## Abstract

We integrated the evidence for evolutionary and plastic trait changes *in situ* in response to climate change in freshwater invertebrates (aquatic insects and zooplankton). The synthesis on the trait changes in response to the expected reductions in hydroperiod and increases in salinity indicated little evidence for adaptive, plastic, and genetic trait changes and for local adaptation. With respect to responses to temperature, there are many studies on temporal trait changes in phenology and body size in the wild that are believed to be driven by temperature increases, but there is a general lack of rigorous demonstration whether these trait changes are genetically based, adaptive, and causally driven by climate change. Current proof for genetic trait changes under climate change in freshwater invertebrates stems from a limited set of common garden experiments replicated in time. Experimental thermal evolution experiments and common garden warming experiments associated with space-for-time substitutions along latitudinal gradients indicate that besides genetic changes, also phenotypic plasticity and evolution of plasticity are likely to contribute to the observed phenotypic changes under climate change in aquatic invertebrates. Apart from plastic and genetic thermal adjustments, also genetic photoperiod adjustments are widespread and may even dominate the observed phenological shifts.

## Introduction

For populations to persist under climate change, they have to deal *in situ* with the temperature increase and associated ecological challenges such as changes in predation rates (De Block et al. 2013). It is unclear to what extent organisms evolve *in situ* to deal with climate change rather than rely on plastic trait changes to buffer them against fitness losses (Hoffmann and Srgò 2011; Merilä 2012; Merilä and Hendry 2013). Reviews made so far suggest plastic responses prevail over genetic responses (Gienapp et al. 2008; Donnelly et al. 2012). Yet, plastic mechanisms may be energetically too costly in the long term (Seebacher et al. 2005) and may not provide the means for populations to continually track changing climatic conditions, for which evolutionary responses are required (Phillimore et al. 2010; Donnelly et al. 2012; Merilä 2012).

The *in situ* persistence of populations under climate change may not only require thermal evolution and thermal plasticity, but also photoperiod adjustments as organisms will have to differently interpret photoregimes and will have to adjust the way they react to these to organize their life cycle (Bradshaw and Holzapfel 2008). Photoperiod adjustments may even shield organisms from the temperature increase by allowing them to track their current climate envelope *in situ* by relying on phenological shifts. It has indeed been suggested that responses to recent rapid climate change operate through photoperiod adjustments rather than through thermal adjustments (Bradshaw and Holzapfel 2006; but see Hoffmann and Srgò 2011). This may be more likely in temperate zone ectotherms in which fitness components are typically improved when temperature increases (Deutsch et al. 2008; Table 1), whereas less so in tropical ectotherms living close to their thermal optimum in which selection to increase thermal tolerance is more likely (Somero 2010; Hoffmann and Srgò 2011).

**Table 1:** Example studies showing typical life-history responses of freshwater invertebrates (aquatic insects and zooplankton) to changes in three abiotic conditions expected under climate change: temperature increase, salinity increase, and hydroperiod shortening

Family	Species	Life-history trait	Reference
Temperature increase			
Coenagrionidae	*Ischnura elegans*	BS↓, DR↑, GR↑	Stoks and De Block (2011), Stoks et al. (2012)
Culicidae	*Wyeomyia smithii*	BS↓, DR↑	Ragland and Kingsolver (2007)
Daphniidae	*Daphnia magna*	GR↑	Mitchell and Lampert (2000)
Daphniidae	*Simocephalus vetulus*	DR↑, FE↑	Van Doorslaer et al. (2007)
Lestidae	*Lestes viridis*	BS↓, DR↑, GR↑	De Block and Stoks (2003)
Limnephilidae	*Limnephilus indivisus*	BS↓(♀), DR↑, GR↑	Jannot (2009)
Salinity increase			
Gerridae	*Aquarius paludum*	SV↓, FE↓	Kishi et al. (2009)
Chironomidae	*Chironomus riparius*	SV↓, DR↓, BS↓(♀)	Lob and Silver (2012)
Culicidae	*Aedes aegypti*	GR↓, DR↓, BS↓	Clark et al. (2004)
Daphniidae	*Daphnia pulex*	FE↓, GR↓, PGR↓, SV↓	Bezirci et al. (2012)
Daphniidae	*S. vetulus*	FE↓, GR↓, PGR↓, SV↓	Loureiro et al. (2012, 2013)
Sididae	*Pseudosida ramosa*	SV↓, FE↓	Freitas and Rocha (2011)
Hydroperiod shortening			
Culicidae	*Aedes triseriatus*	BS↓(♀), DR↑(♀)*	Juliano and Stoffregen (1994)
Culicidae	*Aedes vexans*	BS↓, DR↓	Schäfer and Lundström (2006)
Culicidae	*Ochlerotatus sticticus*	BS=, DR↑	Schäfer and Lundström (2006)
Limnephilidae	*Limnephilus externus*	DR↓, GR↓	Jannot et al. (2008)
Limnephilidae	*L. indivisus*	BS↓(♀), DR=, GR=	Jannot (2009)
Lestidae	*L. viridis*	DR↓, GR↓	De Block and Stoks (2005)

BS, body size; DR, development rate; GR, growth rate; FE, fecundity; PGR, population growth rate; SV, survival.

* Only in one of the two experimental runs.

In the present synthesis, we integrate the available evidence for evolutionary and plastic trait changes in response to climate change in freshwater invertebrates (aquatic insects and zooplankton) of lentic systems, a group that has largely been overlooked in several general reviews of the effects of global warming on organisms (e.g., Walther et al. 2002; Parmesan and Yohe 2003). Besides temperature, salinity and hydroperiod are relevant abiotic conditions for freshwater invertebrates that have been associated with climate change (Schindler 2001; Moss 2012). We therefore will start with an overview of what is known with regard to responses of freshwater invertebrates to the latter two conditions. The major part of the present synthesis will, however, deal with the effects of temperature as these have been studied in more detail and explicitly in the context of climate change. In this temperature part, we will first present an overview of observed *in situ* changes in phenology (and associated population densities) and size attributed to climate change. In line with the theme of this issue (Merilä and Hendry 2013), the focus will then be on the methodologies used and the inferences that can be drawn with regard to the genetic versus plastic nature of the phenotypic changes, their adaptive nature, and the evidence that climate change is the selective force driving these responses.

With regard to freshwater insects, we will pay particular attention as to whether the trait changes likely to be shaped by climate change are related to thermal or photoperiod adjustments. Many freshwater insects, such as caddisflies, mosquitoes, and odonates, have a complex life cycle with an aquatic egg stage followed by an aquatic larval stage where growth and development occurs separated by metamorphosis from a terrestrial stage where reproduction occurs (Stoks and Cordoba-Aguilar 2012). Tropical species (below 15° in latitude) show an unregulated life cycle with continuous larval growth during winter and, as photoperiod remains more constant throughout the year, show low responsiveness to photoperiod cues (Bradshaw and Holzapfel 2007). With increasing latitude, insect larvae typically enter diapause and stop growing during the thermally unfavorable winter period; temperate insects thereby rely strongly on photoperiod cues (Bradshaw and Holzapfel 2007, 2008). Related to this, many freshwater insect species show latitudinal patterns in voltinism, the number of generations per year, with the longer winters at high latitudes allowing only one generation per year, while the much more favorable thermal conditions at low latitudes allowing several generations per year (Corbet et al. 2006). This may eventually generate shorter growing seasons per generation, hence higher time constraints to complete the life cycle, at low latitudes (Stoks et al. 2012). As a result, besides thermal selection, time constraints may become an important selective force along latitudinal gradients, and we will consider their contribution in shaping latitudinal gradients in life histories, which is highly relevant when applying space-for-time substitutions.

## Responses of aquatic invertebrates to salinity and hydroperiod

Increased evaporation and changed rainfall patterns are expected to increase salinity levels and to shorten hydroperiods under climate change (Schindler 2001; Moss 2012). In contrast to temperature, effects of salinity and hydroperiod on fitness-related traits have been much less studied in freshwater invertebrates and not in the context of climate change. Yet, both a higher salinity and a shorter hydroperiod are potentially strong selective pressures that may cause local extinctions (Wellborn et al. 1996; Halse et al. 2003; Loureiro et al. 2013).

The typical responses of freshwater invertebrates to increasing salinity levels are reductions in growth rates, development rates, and fecundity and an increase in mortality rates (Table 1). Some of the studies on zooplankton also documented genotype-by-salinity interactions indicating the potential of plasticity to salinity to evolve (Frisch and Weider 2010; Loureiro et al. 2012, 2013). Signals of local adaptation to salinity in zooplankton are, however, not widespread. In the zooplankter *Simocephalus vetulus*, clones obtained in brackish sites had a higher survival, but only during a short-term exposure period at high salinity levels (6 g/L NaCl) (Loureiro et al. 2012), while there was no signal of salinity adaptation during long-term exposure (Loureiro et al. 2013). Similarly, no signal of local adaptation was detected through time in a *Daphnia magna* population inhabiting a shallow lake with periods of different salinity (Ortells et al. 2005).

Shorter hydroperiods pose a time constraint on the development of aquatic invertebrates as they have to reach a desiccation-resistant stage *in situ* or switch to a terrestrial stage before their pond dries up (Wellborn et al. 1996). There is little experimental proof for adaptive plastic responses to pond drying *per se* in freshwater invertebrates, even in species preferring temporary ponds (Table 1). Only in the mosquito *Ochlerotatus sticticus* (Schäfer and Lundström 2006) and in one of the experimental runs of females of the mosquito *Aedes triseriatus* (Juliano and Stoffregen 1994), development was accelerated when water volume decreased. This may tentatively suggest that aquatic insects may be at a special risk when hydroperiods shorten under global warming. Although, besides the lowering of the water levels, concomitant temperature increases may generate an acceleration of life history (Table 1), these plastic life-history responses are not always enough to allow animals to emerge in time (Wellborn et al. 1996). This suggests that, in the absence of any adaptive evolution, any further shortening of hydroperiods due to climate change will likely increase the chances of local population extinctions and species replacements. None of the studies that looked at the effects of hydroperiod did, however, evaluate the potential for evolutionary trait changes.

## Documented phenotypic changes attributed to global warming

Two general *in situ* responses to global warming in aquatic systems have been identified: seasonal shifts in life cycle events, resulting in changed temporal patterns in population densities, and decreases in body size (Daufresne et al. 2009). Warming-related shifts toward earlier spring events are widespread across aquatic invertebrates (reviews in: Hassall et al. 2007; Thackeray et al. 2010; Sommer et al. 2012; Vadadi-Fülöp et al. 2012). There are indications that these phenological shifts are driven by warming as they are stronger during warmer years (e.g., changes in abundance and seasonality in zooplankton in relation to the North Atlantic Oscillation index, George and Hewitt 1999; Straile and Adrian 2000) and stronger in ectotherms than in endotherms (Thackeray et al. 2010), and also occur when temperature is manipulated in outdoor mesocosm studies (reviewed in Winder et al. 2012). Besides earlier spring events, also later fall events, such as later entering winter diapause, have been attributed to global warming (Bradshaw and Holzapfel 2001; Harada et al. 2005, 2011; Urbanski et al. 2012). While warming-induced phenological shifts may be driven proximally by both thermal and photoperiod responses, so far this only has been explicitly tested and confirmed for photoperiod responses (see below). Yet, thermal adjustments likely also contribute to phenological shifts or the lack of them. For example, seasonal and interannual changes in water temperature are linked to changes in genetic composition of *Daphnia* and associated with genetic differences in thermal tolerance (Ruediger et al. 2012).

Ectotherms typically get smaller at higher temperatures, the so-called temperature–size rule (Atkinson 1994). Recently, the decrease in body size from the community to the individual level has been coined as another general rule regarding the impact of warming on aquatic organisms including zooplankton (Daufresne et al. 2009; MacLennan et al. 2012). At the individual trait level, this has been suggested to partly result from thermal plasticity and interpreted as adaptive responses to a warming climate in the context of Bergmann's rule (Daufresne et al. 2009; Teplitsky and Millien 2013). Yet, warming-induced size shifts do not always occur (e.g., Yvon-Durocher et al. 2011), and the opposite response may be expected because while smaller animals may deal better with long-term increases in mean temperatures, larger animals are expected to deal better with heat waves (Gardner et al. 2011). Note that for both the shifts in phenology and in body size, also other drivers besides global warming may be at work and that the majority of studies do not allow disentangling them. Neither is it known to what extent the changes are genetic, plastic, or a combination (Gardner et al. 2011). Furthermore, the patterns in size and in phenology are not general, and several exceptions have been documented (Winder and Schindler 2004; Vadadi-Fülöp et al. 2012), and the conditions under which the different responses are likely remain elusive.

## Common garden studies replicated in time

**Figure 1:**
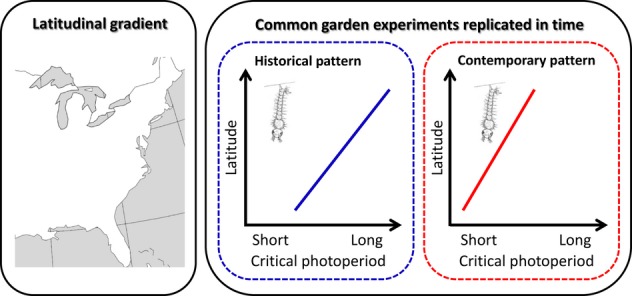
Schematic overview of the common garden experiments across a latitudinal gradient that were replicated in time to demonstrate evolution of the critical photoperiod in two mosquito species (based on Bradshaw and Holzapfel 2001 and Urbanski et al. 2012). The critical photoperiod shows a latitudinal cline increasing with latitude. Comparing the historical pattern with the contemporary pattern indicates that global warming resulted in the evolution of shorter critical photoperiods at a given latitude.

All three studies that have documented changes in phenology in response to climate change in aquatic invertebrates focused on photoperiod responses. In all three species, the North American pitcher plant mosquito *Wyeomyia smithii* (Bradshaw and Holzapfel 2001), the water strider *Aquarius paludum* (Harada et al. 2005, 2011), and the Asian tiger mosquito *Aedes albopictus* (Urbanski et al. 2012), the photoperiod response to warming was demonstrated using common garden experiments that were replicated in time (Fig. 1, Table 2). The critical photoperiod (the day lengths that result in the commencement of diapause) when tested many years later (5 and 23 years in *W. smithii*, 5 and 11 years in *A. paludum*, 20 years in *A. albopictus*) shifted toward shorter photoperiods corresponding with later dates, which is consistent with an adaptive response to longer growing seasons and therefore with the effects of global warming on seasonality.

**Table 2:** Summary of studies on freshwater invertebrates (aquatic insects and zooplankton) designed to examine plastic and genetic responses of traits driven by climate change

Family	Species	Trait type	Genetic	Plastic	Adapt	Cause	Time?	Reference
Common garden experiments replicated in time								
Culicidae	*Aedes albopictus*	CP	Y(2,5)	.	.	.	EX	Urbanski et al. (2012)
Culicidae	*Wyeomyia smithii*	CP	Y(2,5)	.	Y(1)	Y(1)	EX	Bradshaw and Holzapfel (2001), Bradshaw et al. (2004)
Gerridae	*Aquarius paludum*	CP	Y(2)	.	.	Y(1)	EX	Harada et al. (2005, 2011)
Experimental thermal evolution								
Daphniidae	*Daphnia magna*	CS*, PGR*, PL*	Y(2,4)	Y(2,3)	Y(1)	Y(3)	.	Van Doorslaer et al. (2009a)
Daphniidae	*D. magna*	CS	Y(2,4)	Y(2,3)	Y(1)	Y(3)	.	Van Doorslaer et al. (2009b)
Daphniidae	*D. magna*	BS, PL	Y(2,4)	Y(2,3)	Y(1)	Y(3)	.	Van Doorslaer et al. (2010)
Daphniidae	*Simocephalus vetulus*	AR, FE, SV	Y(2,4)	Y(2,3)	Y(1)	Y(3)	.	Van Doorslaer et al. (2007)
Space-for-time substitutions								
Coenagrionidae	*Ischnura elegans*	BT, DR, GR, PY, PT	Y(2,5)	Y(2,3)	Y(4)	Y(1)	.	Shama et al. (2011), Stoks and De Block (2011), Stoks et al. (2012), De Block et al. (2013), Dinh Van et al. (2013)
Libellulidae	*Orthetrum cancellatum*	CP, GR	Y(2,5)	.	.	Y(1)	.	Flenner et al. (2010)
Chironomidae	*Chironomus riparius*	PGR, FE, PT	Y(2,5)	Y(2,3)	Y(4)	Y(1)	.	Nemec et al. (2013)
Culicidae	*W. smithii*	TT	Y†/N(2,5)	.	Y(1)	Y(1)	.	Bradshaw et al. (2000, 2004), Zani et al. (2005), Ragland and Kingsolver (2008)
Culicidae	*W. smithii*	DR, PT	Y(2,5)	Y(2,3)	.	Y(1)	.	Ragland and Kingsolver (2007)
Daphniidae	*D. magna*	GR	N(2,5)	Y(2,3)	.	.	.	Mitchell and Lampert (2000), Chopelet et al. (2008)
Daphniidae	*D. magna*	PY	Y(2,5)	Y(2,3)	.	.	.	Chopelet et al. (2008)
Daphniidae	*Daphnia pulex*	TT	Y(2,5)	Y(2,3)	.	.	.	Williams et al. (2012)

Trait type (type of trait examined): AR, age at reproduction; BT, behavioral trait; CP, critical photoperiod to induce diapause; CS, competitive strength; DR, development rate; FE, fecundity; GR, growth rate; PGR, population growth rate; PL, plasticity in the traits; PY, physiological trait; SV, survival; TT, Thermal tolerance. A ‘Y’ indicates that evidence was found for genetic or plastic responses in traits or that the adaptive nature or causality was investigated; ‘N’ indicates that evidence was not found; ‘.’ indicates that it was not investigated. Numbers next to a ‘Y’ or ‘N’ denote the method of investigation invoked (numbering based on Merilä and Hendry 2013). Genetic categories: 1, animal models; 2, common garden studies; 3, comparison to model predictions; 4, experimental evolution; 5, space-for-time substitution; 6, molecular genetic approaches; Plastic categories: 1, animal models; 2, common garden studies; 3, experimental studies; 4, fine-grained population responses; 5, individual plasticity in nature; Adapt categories: 1, reciprocal transplants; 2, phenotypic selection estimates; 3, genotypic selection estimates; 4, *Q*_ST-_*F*_ST_; Cause categories: 1, common sense; 2, phenotype by environment interactions; 3, experimental selection/evolution; For full descriptions of all categories, see Merilä and Hendry (2013). Time? (time component included in data collection): EX, common garden experiment through time.

* Only in the treatment where recurrent periods of exponential growth were possible.

† Only when populations were stressed to the brink of extinction or when the adverse effects of temperature could accumulate over several generations in the experiment (Bradshaw et al. 2004).

The magnitude of this shift toward shorter critical photoperiods tended to be stronger at higher latitudes in *W. smithii*, and across latitudes, it was estimated to be 0.25 h (1972 vs 1996) and 1.02 h (1988 vs 1993) (Bradshaw and Holzapfel 2001). At the mean latitude of 35°N, the shift was 0.50 h (1988 vs 2008) in *A. albopictus* (Urbanski et al. 2012), and at the single latitude studied, it was 0.5 h (1991 vs 2002) in *A. paludum* (Harada et al. 2005, 2011). To give an idea about how shortened critical photoperiods translate in the number of days that animals enter diapause later: at 50°N latitude, the critical photoperiod declined from 15.79 to 15.19 h from 1972 to 1996 in *W. smithii*, which corresponds to 9 days later in the fall of 1996 than 1972 (Bradshaw and Holzapfel 2001). This is similar to the advancement of other seasonal events in the temperate region over the same time span in birds (Charmantier and Gienapp 2013) and frogs (Urban and Phillips 2013).

The three studies differ in the strength of inference with respect to the genetic nature of the changes, as well as to the degree in which global warming has been demonstrated to be the selective agent behind the shifts. While *A. albopictus* was kept in the laboratory for 3–14 generations before being tested, for the other two species, field-collected (*W. smithii*) and F1 larvae (*A. paludum* and *W. smithii*) were used, making the inference about genetic changes less strong. While the latter studies did not use F2 larvae, for *W. smithii*, it was, however, argued that any bias resulting from not having fully excluded the contribution of environmental effects from the field shaping the photoperiod response would be against showing a genetic change (Bradshaw and Holzapfel 2001). While these changes in photoperiod response strongly indicate responses to ongoing climate change in *W. smithii* and *A. paludum*, this interpretation is somewhat less straightforward in *A. albopictus* whose North American populations were tested the first time only a couple of years after the invasion of this continent. Therefore, the observed genetic shifts may represent continued adaptation of invasive populations to local climate conditions rather than tracking of ongoing contemporary climate warming (Urbanski et al. 2012). Yet, in any case, the study shows the potential of this species to locally adapt to climate change.

## Experimental thermal evolution

**Figure 2:**
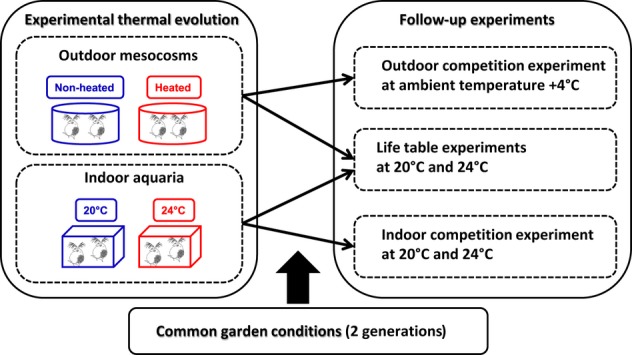
Schematic overview of the experimental thermal evolution trials and associated follow-up experiments to demonstrate the evolutionary potential of zooplankton species to respond to global warming. These studies consisted of three steps. In the first step, animals were exposed to control and warming conditions for several generations. Two outdoor mesocosm studies were run under nonheated (ambient temperatures) conditions and under following heated conditions: IPCC scenario A2 and A2 + 50% (Van Doorslaer et al. 2007) and IPCC scenario A1FI (Van Doorslaer et al. 2009b, 2010). One indoor aquarium study was run at 20 and 24°C (Van Doorslaer et al. 2009a). In the second step, experimental animals obtained from step 1 were kept for at least two generations under common garden conditions to minimize interference from maternal effects and acclimation to allow testing the genetic basis of changes in trait means and their thermal plasticity in the next step. In the third step, the evolutionary response was tested in a series of follow-up experiments: life table experiments with treatments differing 4°C where life-history traits were studied in detail, and competition experiments where the fitness consequences of thermal evolution were tested in terms of changed competitive strength. All four studies showed signals of thermal evolution with regard to the means of life-history traits (e.g., age at first reproduction, body size, offspring number), and one study also with regard to thermal plasticity (Table 2).

A limited number of studies on aquatic invertebrates have relied on experimental evolution to directly evaluate the potential for an evolutionary response under thermal selection mimicking IPCC warming scenarios (Table 2). Linked to their short generation times, ease of handling, and the presence of clonal lineages, all these four studies were conducted on zooplankton species. After several generations of thermal selection in outdoor mesocosms or indoor aquaria, these studies reared animals for at least two generations under common garden conditions to minimize interference from maternal effects and acclimation (Fig. 2). These are important precautionary steps as acclimation effects can be strong. All four studies documented genetic changes in the means of some of the studied life-history traits under warming (e.g., age at first reproduction, body size, offspring number), and one study also documented genetic changes in thermal plasticity (Table 2).

A drawback of experimental evolution trials is that the adaptive nature of the documented evolutionary trait changes cannot always simply be deduced from the direction of the trait change. For example, for body size, the evolutionary increase was opposite the plastic decrease with increasing temperature, suggesting that the microevolutionary response compensated for temperature-induced plasticity (Van Doorslaer et al. 2010). To rule out the possibility of neutral or maladaptive evolution, the adaptive value of the genetic trait changes was evaluated by carrying out competition experiments to obtain a more integrated measure of adaptation to different temperature regimes (cf. Bennett et al. 1992). Eventually, in all four studies, experimental trait evolution could be interpreted as adaptive as at higher temperatures, it increased survival (Van Doorslaer et al. 2007), or increased competitive strength against nonheat-adapted genotypes (Van Doorslaer et al. 2009a) or against invading southern preadapted genotypes (Van Doorslaer et al. 2009b, 2010), thereby also illustrating that thermal microevolution may impact ecological interactions.

All experimental evolution trials on zooplankton confirmed that thermal evolution can be rapid (within months). This may be related to the fact that these microevolutionary responses were fueled by standing genetic variation rather than driven by *de novo* mutations (Barrett and Schluter 2008). On the one hand, evolution in nature may even go faster because in the experiments, only a small subset of the available genotypes in the natural populations were present. On the other hand, global warming will occur gradually and therefore may generate slower responses that may differ from evolutionary responses to the sudden temperature changes applied in the experimental evolution trials (see Collins et al. 2007). These case studies therefore indicate the potential for rapid evolutionary shifts to warming, but do not allow for precise predictions of the specific features of the evolutionary responses to be expected in the wild.

Another complicating factor that may make it difficult to generate precise predictions is that in nature, selection pressures and the resulting evolutionary response may be strongly context dependent. Several context-driven complexities associated with thermal trait evolution were revealed in these studies. First, the indoor aquarium experiment showed that the evolutionary response to thermal selection may strongly depend on population dynamics: only in the treatment where recurrent periods of exponential growth were possible, a signal of thermal evolution was recovered (Van Doorslaer et al. 2009a). Second, comparing the indoor aquarium experiment with the outdoor mesocosm experiment where *Daphnia* were embedded in a natural community including interspecific competitors, predators, and parasites highlighted the importance of the ecological context for the outcome of thermal evolution (De Meester et al. 2011). While thermal microevolution of individual performance and not of size at maturity occurred in the indoor aquarium experiment, in the outdoor mesocosm experiment, the opposite pattern occurred (Van Doorslaer et al. 2009a, 2010). This context dependency highlights the complexity of predicting evolutionary responses based on experiments carried out in simplified environments (Reznick and Ghalambor 2005). While microevolutionary responses in these experimental evolution trials can be unambiguously ascribed to temperature as selective agent, in the more complex seminatural setting of the mesocosm experiment, these responses may be caused by direct selective effects of temperature as well as by indirect temperature-mediated effects via changes in interactions with other species. Yet, despite the fact that the indoor aquarium experiments and outdoor mesocosm experiments were run under widely different environmental conditions, the key observation of a rapid evolutionary response to the temperature change was observed under both settings. These experiments thus point to the generality of the capacity of local populations of aquatic invertebrates to respond genetically to a sudden change in temperature over a short interval of time (De Meester et al. 2011).

## Latitudinal clines and space-for-time substitutions

**Figure 3:**
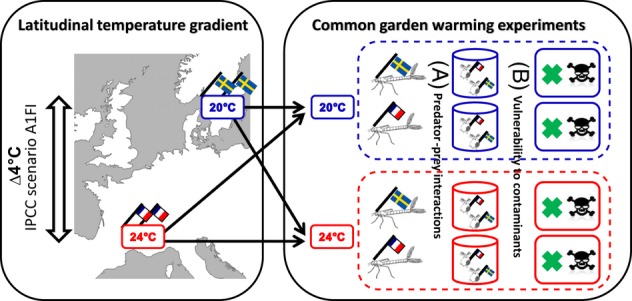
Schematic overview of the space-for-time substitution approach to simulate the effects of global warming on (A) predator–prey interactions and (B) vulnerability to contaminants (based on De Block et al. 2013 and Dinh Van et al. 2013). Damselfly larvae from replicated low-latitude (French) and high-latitude (Swedish) populations were reared in common garden experiments from the egg stage at 20 and 24°C, reflecting the mean summer water temperatures at high and low latitudes, respectively. The spatial 4°C temperature difference matches the predicted temperature increase at the high latitude by 2100 according to IPCC scenario A1FI (IPCC 2007). Therefore, the responses of the low-latitude larvae reared at 24°C are relevant proxies for the responses to be expected under gradual long-term thermal evolution in high-latitude larvae currently living at 20°C. Note that the original design of both studies also included animals from central latitudes.

Few studies in aquatic invertebrates have explicitly explored the possible effects of global warming on trait changes using a space-for-time substitution approach along a latitudinal gradient (Fig. 3, Table 2). With the exception of the two above-mentioned mosquito studies (Bradshaw and Holzapfel 2001; Urbanski et al. 2012), this approach was not used as a complement to assist inferences about the genetic nature of changes observed in the wild at a given location (Merilä and Hendry, 2013). Instead, this approach has been mainly applied to generate plausible scenarios on how future warming may change traits in northern populations based on current phenotypes in southern populations already experiencing the higher temperatures (cf. Fukami and Wardle 2005).

Latitudinal patterns in the photoperiod response of diapause induction in the mosquitoes *A. albopictus* and *W. smithii* showed a genetic cline in critical photoperiod, from short in southern populations to long in northern populations (Fig. 1). This matches the earlier onset of winter at northern latitudes, hence the onset of diapause at longer day lengths (Bradshaw and Holzapfel 2008), and therefore represents local adaptation to latitudinal variation in the length of the favorable growth season. These latitudinal clines suggested that under global warming, northern populations should shorten their critical photoperiod, which was upheld in the common garden experiments (Bradshaw and Holzapfel 2001; Urbanski et al. 2012). Moreover, in a companion study, it was convincingly shown that a mismatch to the local photoregime along a latitudinal gradient may drastically reduce fitness (Bradshaw et al. 2004). This provided extra support to the inference that the temporal genetic changes in the photoperiod response are adaptive and driven by global warming. Noteworthy, in *A. albopictus*, it was explicitly shown that in contrast to the photoperiod response, size did not show any latitudinal patterns indicating that climate-mediated selection on body size is not general (Urbanski et al. 2012).

Latitudinal differences in the photoperiod response have also been observed for diapause induction in water striders (Blanckenhorn and Fairbairn 1995) and for growth and development rates in larval odonates (Flenner et al. 2010; Sniegula and Johansson 2010; Sniegula et al. 2012). Although these studies could not exclude a contribution of maternal effects, they tentatively suggest that a genetic change in the photoperiod response to warming may be relatively common in aquatic insects. Note that this may contribute to the observed latitudinal shifts in phenology (e.g., Johansson et al. 2010) and is thus in line with the temporal phenological shifts observed under global warming for odonates (Hassall et al. 2007).

Intraspecific latitudinal differences in thermal adaptation of life-history traits have been documented in many species (Schilthuizen and Kellermann 2013; Urban and Phillips 2013), but rarely in aquatic invertebrates (see below). This has fueled the idea that global warming will not typically generate genetic changes in response to higher temperatures *per se* (Bradshaw and Holzapfel 2006). In one of the best studied species in this context, the mosquito *W. smithii*, thermal tolerance both measured as heat knockdown times (Ragland and Kingsolver 2008) and as survival and an integrated fitness measure (year-long cohort replacement rate) indeed did not show a latitudinal pattern (Bradshaw et al. 2000; Zani et al. 2005). In the water flea *Daphnia pulex*, southern clones showed a higher heat tolerance than northern clones when tested at low and intermediate, but not at high acclimation temperatures, which may suggest that phenotypic plasticity may dominate in shaping thermal tolerance under gradual climate change (Williams et al. 2012). In the isopod *Asellus aquaticus,* heat tolerance was higher in a southern than in a northern population (di Lascio et al. 2011), yet maternal effects were not controlled for in this study.

Also with regard to other life-history traits, such as growth and development rates, signals for local thermal adaptation along a latitudinal gradient are few and relatively small in freshwater invertebrates. Studies on the water flea *D. magna* could not detect a signal of local thermal adaptation in life history along a latitudinal gradient (Mitchell and Lampert 2000; Chopelet et al. 2008). This lack of thermal response has been suggested to be driven by latitudinal patterns in the timing of the growing season, so that thermal regimes experienced during the growing season do not differ much across latitudes (Mitchell and Lampert 2000). Yet, oxygen consumption rates did show local thermal adaptation being higher in the northern clones at the low temperature and higher in the southern clones at the high temperature (Chopelet et al. 2008). Moreover, within a given *D. magna* population, thermal specialist clones may occur that are adapted to specific parts of the growing season (Carvalho 1987; Ruediger et al. 2012).

In freshwater insects, the presence of a strong signal of thermal adaptation along latitudinal gradients seems to depend on the latitudinal pattern in voltinism. In the midge *Chironomus riparius* that has multiple generations across its entire latitudinal range, growth rates decreased going from 20 to 28°C, but much less so in warmer, southern populations (Nemec et al. 2013), indicating a clear pattern of local thermal adaptation. However, latitudinal gradients in thermal adaptation have not been detected or were subtle in the freshwater insects with strong changes in voltinism (damselflies: Stoks et al. 2012; dragonflies: Flenner et al. 2010; mosquitoes: Ragland and Kingsolver 2008; water striders: Blanckenhorn and Fairbairn 1995). In the latter species, southern populations have more generations per year, and as a result have less time per generation and evolve faster life histories (growth rates and development rates) than northern populations, which have one generation per year or per 2 years. In such conditions, life-history differences among latitudes seem to be primarily driven by changes in voltinism. In the mosquito *W. smithii,* there is some indication for thermal adaptation across a latitudinal gradient using an integrated fitness measure, the year-long cohort replacement rate across all four seasons, but only when populations were stressed to the brink of extinction or when the adverse effects of temperature could accumulate over several generations in the experiment (Bradshaw et al. 2004). Furthermore, it was shown that southern *W. smithii* accelerate development more at higher temperatures than northern animals (Ragland and Kingsolver 2007). Similarly, in the damselfly *Ischnura elegans,* southern animals accelerate growth more at higher temperatures than northern animals (Shama et al. 2011). A *Q*_ST_-*F*_ST_ comparison indicated that the latitudinal differentiation in this damselfly was likely adaptive and driven by selection rather than being caused by genetic drift (but see Edelaar et al. 2011 for a cautionary note on this method). Furthermore, in each population, there was genetic variation for both the slope and the intercept of thermal reaction norms, suggesting potential for future adaptation (Shama et al. 2011). This suggests that under global warming, these insects likely will shift toward more generations per year at a given site (as, for example, shown in water striders, Harada et al. 2011), and to compensate for the resulting shorter growing periods per generation will likely evolve a faster life history. Given that southern populations have faster life histories suggests that they may be superior competitors when invading northern sites, and thus replace the northern genotypes unless northern populations can adjust rapidly enough to the changing thermal regime (Shama et al. 2011).

**Figure 4:**
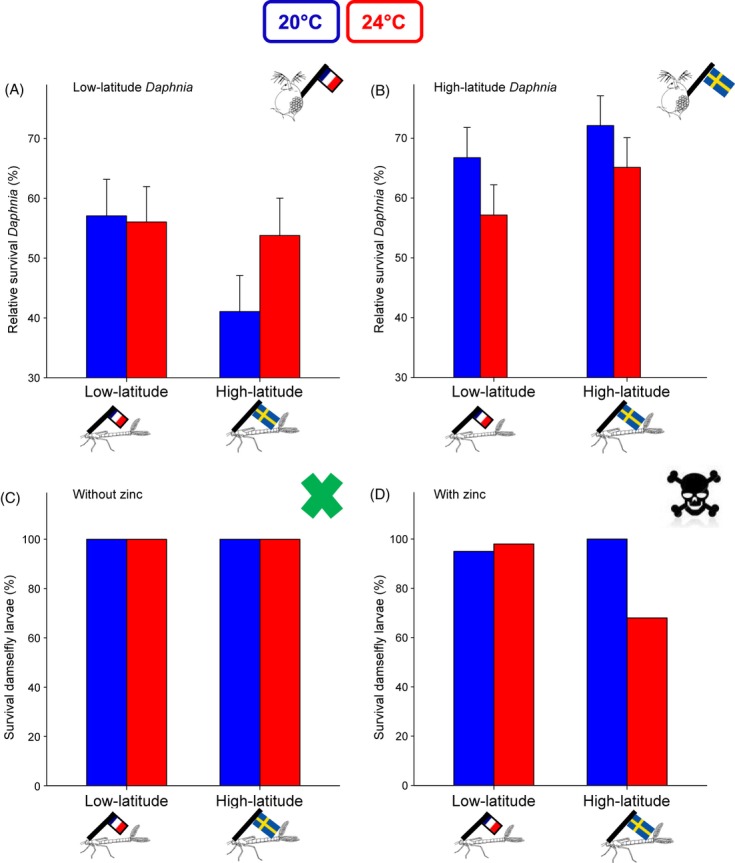
Summary of the results obtained in the common garden warming experiments illustrated in Fig. 3 (after De Block et al. 2013 and Dinh Van et al. 2013). (A–B) The relative survival of the *Daphnia* prey of a given latitude was higher at its latitude-specific temperature than at the other temperature, unless the *Daphnia* are confronted with the local damselfly predators. This indicates local thermal adaptation of both prey and predators. (C–D) While at 20°C, all damselfly larvae from both latitudes survived when exposed to zinc, at 24°C, survival was reduced in the high-latitude larvae exposed to zinc, but not in the low-latitude larvae. This indicates that thermal adaptation of the low-latitude larvae buffered against the increased toxicity of zinc at the higher temperature.

Two recent studies on the damselfly *I. elegans* relied on the space-for-time substitution approach to demonstrate the potential of gradual thermal evolution to mitigate the effects of global warming on predator–prey interactions (De Block et al. 2013) and on the vulnerability to contaminants (Dinh Van et al. 2013). In both studies, animals were reared under common garden conditions reflecting the mean summer water temperatures in ponds in northern (20°C) and southern (24°C) Europe with the temperature difference reflecting the predicted temperature increase under IPPC scenario A1FI (IPCC 2007) (Fig. 3). The first study showed local thermal adaptation in both the *I. elegans* predators and the *D. magna* prey (De Block et al. 2013). While survival was higher at 20°C than at 24°C in high-latitude prey, and higher at 24°C than at 20°C in low-latitude prey, this was only true when confronted with damselfly predators from other latitudes and not with predators adapted to the local thermal conditions (Fig. 4A–B). The second study showed that the used zinc concentrations were not lethal at 20°C, yet mortality occurred when high-latitude, but not low-latitude damselfly larvae were exposed to zinc at 24°C, indicating local thermal adaptation in the low-latitude larvae buffering against the increased toxicity of zinc at 24°C (Dinh Van et al. 2013) (Fig. 4C–D). The likely reason for this pattern was that low-latitude larvae had higher amounts of energy to defend against and repair damage as high-latitude larvae showed a much stronger zinc-induced reduction of food intake at 24°C. The followed space-for-time substitution approach in both studies suggested the invasion success of northward moving predators and prey to be strongly latitude-specific and to depend upon the level of contaminants in the aquatic habitat.

## Conclusions

A general observation is that in aquatic invertebrates, there is a strong discrepancy between field observations and experimental-evolutionary work on trait changes under global warming. On the one hand, there are many observational studies on temporal changes in phenology and body size in the wild that are believed to be driven by global warming. Yet, we lack a rigorous demonstration whether these trait changes are genetically based, adaptive, and causally driven by global warming. On the other hand, nearly all (of the few) experimental studies inferring that particular traits have the potential to evolve in an adaptive way under global warming lack information on the realized temporal changes in these traits in the wild. A notable exception was the common garden studies that were replicated in time on mosquitoes (Bradshaw and Holzapfel 2001; Urbanski et al. 2012) and water striders (Harada et al. 2005, 2011). The latter studies provide the only case of strong inferences about documented evolutionary change in response to global warming in the wild in aquatic invertebrates. These studies showed photoperiod rather than thermal adjustments to global warming. Yet, although the observations are in line with expectations based on common sense, in *A. albopictus,* it is not entirely sure that global warming was the selective agent (Urbanski et al. 2012). The lack of evidence of genetic changes in response to global change must, however, not necessarily be interpreted as suggesting the absence of such a response. Rather, few studies tested for genetic changes and the adaptive nature of the observed responses. As a matter of fact, most experimental studies provide evidence for evolutionary potential.

Of the toolbox of six approaches for inferring genetic change (Merilä and Hendry 2013), two experimental approaches have been applied in aquatic invertebrates, but without information on temporal trait changes in the field. In a first approach, experimental evolution trials convincingly demonstrated adaptive genetic change in life-history traits in response to warming in zooplankton (Van Doorslaer et al. 2007, 2009a,b, 2010). In a second, space-for-time substitution approach, studies on aquatic insects and zooplankton documented thermal adaptation or adaptation to the local photoperiod along a latitudinal gradient using common garden experiments. These two approaches do, however, merely serve as ‘proof-of-principle’ that genetically based trait change is possible (Merilä and Hendry 2013). These approaches provide a first attempt of a framework to understand and predict temporal trait changes *in situ* and to generate scenarios about what may happen in northern populations both in the absence and in the presence of invasion of southern genotypes and interacting species.

The few experimental studies that allowed testing for it indicated genetic change probably occurred (Bradshaw and Holzapfel 2001; Harada et al. 2005, 2011; Urbanski et al. 2012) or has the potential to occur (Van Doorslaer et al. 2007, 2009a,b, 2010). This is not unexpected as rapid evolutionary responses to climate change have been reported in long-lived vertebrates (Boutin and Lane 2013; Urban and Phillips 2013). The relatively short generation length of freshwater invertebrates may facilitate such evolutionary tracking of the changing climate. Yet, even if evolution in response to warming would be widespread, this is no guarantee that evolution will be rapid enough to keep pace with global warming and allow *in situ* persistence (Etterson and Shaw 2001). Moreover, genetic tracking of the new temperature regimes by a given species may not be enough, as species are embedded in communities, and it is unlikely that all biotic interactors will evolve at the same speed, which may result in the disruption of ecological interaction networks (e.g., through phenological mismatches, Winder and Schindler 2004). Also, there may be costs of evolution involved, where adaptation to one stressor results in a weakening of the resulting genotypes in their response to other stressors (e.g., Jansen et al. 2011). The limited evidence suggests that besides genetic changes, also phenotypic plasticity and evolution of plasticity likely will contribute to the observed phenotypic changes under global warming in aquatic invertebrates. The current evidence also indicates that besides thermal adjustments, also photoperiodic adjustments to global warming are widespread and may even dominate the phenological shifts. Finally, we wish to stress the fact that adaptation to climate change is a much more complex challenge than a simple response to increasing temperatures, as it also involves adapting to changes in predator regime (e.g., De Block et al. 2013), in the toxicity of contaminants (e.g., Dinh Van et al. 2013), in community composition, and in interaction networks between competitors, predators, and parasites (e.g., Hall et al. 2006).

## Future research directions

The present synthesis highlighted the fact that, as is the case in many other taxa (Merilä and Hendry 2013), more rigorous tests to infer genetic change in response to global warming are needed in freshwater invertebrates. This is especially true for responses to the expected increases in salinity and the shortening of the hydroperiods under climate change, where tests are missing. Of the toolbox of six approaches for inferring genetic change (Merilä and Hendry 2013), two methods have remained largely unexplored in this group. Comparisons to predictions from multivariate evolution models and molecular genetic approaches should be possible to apply in aquatic invertebrates. Obviously, these approaches should ideally be combined and applied to study species with well-documented temporal changes in phenology and body size in the wild.

A particularly promising methodology is to replicate common garden experiments on freshwater invertebrate populations through time using resurrection ecology (as has been applied in plants, Franks et al. 2013). While many freshwater invertebrates have dormant egg banks that have been exploited to reconstruct microevolutionary changes in response to anthropogenic stressors (Jeppesen et al. 2001), up to now, there are no published studies exploiting these archives in replicated common garden experiments to study genetic adaptation driven by global warming. By comparing representative samples from different time periods and when paying attention to avoid working with biased population samples, this approach can be applied to reconstruct responses to warming (Angeler 2007). Moreover, when tested in common garden experiments, they also allow correcting for maternal effects to infer the possible genetic basis for the responses (De Meester et al. 2011). To infer whether adaptive changes have occurred, such a method could be combined with two other techniques: (i) the observed responses could be compared with the predicted responses generated by evolutionary models (cf. Etterson and Shaw 2001) and (ii) the fitness of the resulting phenotypes could be evaluated using reciprocal transplants between the temporally separated temperature regimes (cf. Van Doorslaer et al. 2009a). Such resurrection approach cannot only be used to reconstruct historical phenotypic traits, but also to reconstruct adaptation at the genomic level (paleogenomics; e.g., Orsini et al. 2013; Pauls et al. 2013). A particularly strong approach to provide solid evidence of historical selection and adaptation at the genome level would be to use a combined genome scan approach incorporating not only temporal gradients using resurrection ecology, but also well-characterized spatial thermal gradients and experimental thermal evolution trials so as to triangulate the genetic basis of thermal adaptation and to identify and monitor candidate genes linked to warming (for an example on other stressors, see Orsini et al. 2012).

With regard to the stressors relevant to consider in the context of climate change, we see two important directions for future research on aquatic invertebrates. First, nearly all studies included in present synthesis used constant temperatures and did not consider the effect of a heat wave (but see e.g., Zani et al. 2005). Yet, future climate will be characterized in many regions by increases in the frequency of extreme temperature events (IPCC 2012) whose effects cannot be just extrapolated from regular warming experiments (Thompson et al. 2013). Second, because of the potential interactions of climate with other large-scale environmental stressors such as UV-B irradiance, eutrophication, and pollution, future studies need to consider plastic and evolutionary responses to multiple stressors (Keller 2007; Moss 2012). This is especially needed as it is becoming increasingly clear that anthropogenic stressors often reinforce each other (Darling and Côté 2008) and the ability of organisms to adapt to single stressors associated with climate change may underestimate their effective potential to persist in a world with cocktails of anthropogenic stressors.
